# Effects of porcine reproductive and respiratory syndrome virus (PRRSV) on thyroid hormone metabolism in the late gestation fetus

**DOI:** 10.1186/s13567-022-01092-3

**Published:** 2022-09-30

**Authors:** Erin K. Ison, Amber S. Hopf-Jannasch, John C. S. Harding, J. Alex Pasternak

**Affiliations:** 1 Department of Animal Science, Purdue University, West Lafayette, IN 47906 USA; 2 Bindley Bioscience Center, Purdue University, West Lafayette, IN 47906 USA; 3 Department of Large Animal Clinical Sciences, Western College of Veterinary Medicine, University of Saskatchewan, 52 Campus Dr., Saskatoon, SK S7N 5B4 Canada

**Keywords:** PRRS, host–pathogen interaction, maternal, fetal, hypothyroidism, thyroid hormone

## Abstract

Porcine reproductive and respiratory syndrome virus (PRRSV) in late gestation causes a profound suppression of circulating maternal and fetal thyroid hormone during a critical window of development. To understand this relationship, we evaluated thyroid hormone metabolism at the maternal–fetal interface and within fetal tissues, along with hormone metabolite levels in serum. Fetuses were classified using an established model based on viral load in serum and thymus, and preservation status, including uninfected (UNIF), high-viral load viable (HV-VIA), and high-viral load meconium-stained (HV-MEC), with additional controls from sham-inoculated gilts (CON). Expression of three iodothyronine deiodinases, five sulfotransferases, sulfatase, and two solute carriers known to transport thyroid hormone were evaluated in maternal endometrium and fetal placenta, liver, and kidney. Serum thyroxin (T4), reverse triiodothyronine (rT3), and diiodothyronine (T2) were evaluated via liquid chromatography tandem mass spectrometry. Significant changes in gene expression were observed in all four tissues, with the liver being the most severely impacted. We observed local and fetal specific regulation of maternal tissues through significant upregulation of DIO2 and DIO3 expression in the endometrium corresponding to infected but viable fetuses relative to uninfected and control fetuses. Expression levels of DIO2 and DIO3 were significantly higher in the resilient (HV-VIA) fetuses relative to the susceptible (HV-MEC) fetuses. A substantial decrease in serum T4 was confirmed, with no corresponding increase in rT3 or T2. Collectively, these results show that thyroid hormone metabolism is altered at the maternal–fetal interface and within the PRRSV infected fetus and is associated with fetal viability.

**Supplementary Information:**

The online version contains supplementary material available at 10.1186/s13567-022-01092-3.

## Introduction

Porcine reproductive and respiratory syndrome virus (PRRSV) is a devastating and highly transmissible virus that results in annual losses within the United States swine industry in excess of $660 million [1]. The virus is especially concerning within breeding herds because it can cross the typically limiting epitheliochorial placenta of the pig during late gestation and increase the number of weak and aborted piglets [2]. After an established viral infection, the fetus exhibits a robust immune response [3–5], disruption in developing organs, including delays in heart maturation, and suppressed thyroid gland function [6, 7]. Advanced infection is indicated in susceptible fetuses by the expulsion of the fetal digestive contents, or meconium, which stains the fetal body and indicates early compromise [8].

The preservation and viral load status of fetuses from PRRSV-infected dams are not consistent within litters. While some fetuses demonstrate complete resistance, with no detectable viral load (UNIF), others from the same litter have extremely high viral loads in both serum and tissue. Fetuses with high viral load can present as severely compromised with meconium-staining on head and/or body (HV-MEC) or have a normal physical appearance and minimal signs of infection (HV-VIA) [9]. The variation in fetal preservation following PRRSV infection suggests that fetuses with resistance (UNIF) or resilience (HV-VIA) to the virus are physiologically different from those that are susceptible (HV-MEC), which are expected to die in-utero in pregnancies continuing to parturition (gestation day 115). The underlying cause of this variation in phenotype is not well understood but is unlikely to result from the disruption of a single organ [6]. The comparison of dysregulations in systems critical for fetal development across the resistant (UNIF), resilient (HV-VIA), and susceptible (HV-MEC) fetuses could uncover a way to mitigate fetal response to PRRSV infection.

**Figure 1:**
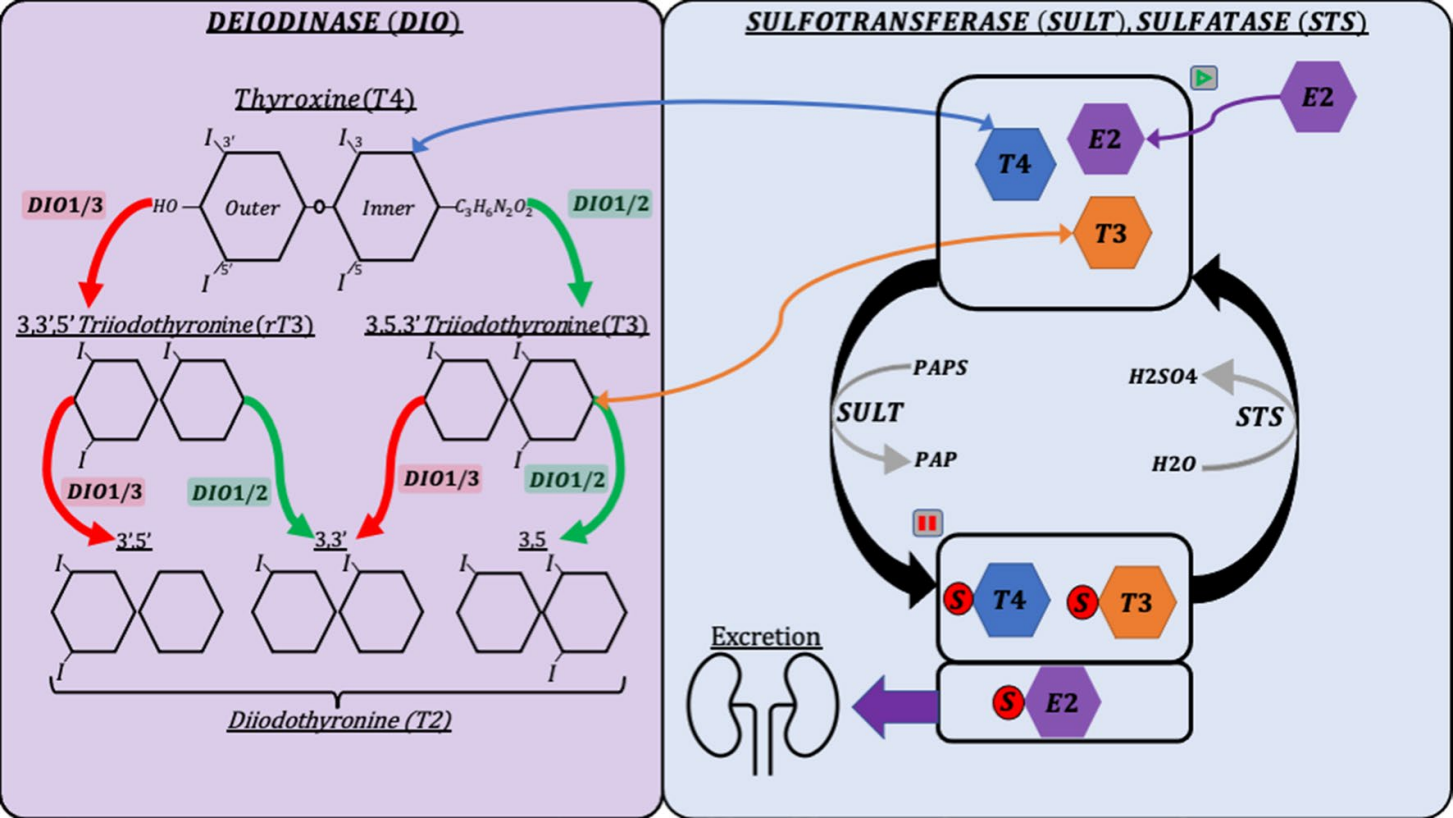
**Graphical representation of the relationship between genes in the deiodination and sulfation pathways.** Green arrows represent hormone activation and red arrows represent hormone deactivation. Blue and orange arrows represent the movement of corresponding thyroid hormones. Purple arrows represent the movement of estradiol (E2) as an additional example of hormones that can enter the sulfation pathway. Grey arrows represent enzymatic activity. Pause and play symbols represent bioactivity dormancy and resumption, respectively.

The various thyroid hormones share a common base structure of two rings (inner and outer) and are distinguished from one another by the presence of iodine molecules at four sites [10]. The bioactivity of the hormones depends on the number and positions of these iodine molecules, which can be sequentially removed through enzymatic reactions [11]. Deiodinases are enzymes that activate and deactivate thyroid hormones by removing iodine from one of the amino acid rings (Figure 1). DIO1 can remove iodine from both rings, while DIO2 and DIO3 are restricted to the outer and inner rings, respectively. Inner ring deiodination by DIO1 or DIO3 deactivates thyroid hormone, producing inactive metabolites such as reverse-triiodothyronine (rT3) and variable diiodothyronines (3,3′, 3,5, 3′,5′T2). Outer ring deiodination by DIO1 or DIO2 activates thyroid hormone and is the most common method of producing the bioactive triiodothyronine (T3). To elicit a response within a target tissue, thyroid hormones must be transported across cell membranes, where they subsequently interact with nuclear receptors TRα and TRβ [12]. Thyroid hormones play a fundamental role in regulating overall fetal growth and development of vital organs, such as the heart and brain [13]. Some mammalian fetuses, including humans, rely on the dam to supply the bioactive thyroid hormone needed for development until fetal thyroid autonomy occurs during mid-gestation [14]. However, the fetal pig thyroid gland is productive early in gestation and produces increasing amounts of hormone until parturition [15]. Additionally, an enzymatic barrier to thyroid hormone at the pig placenta significantly limits maternal transfer to the fetus, suggesting that the pig fetus does not rely on supplemental maternal hormone [16].

Homeostasis of the hypothalamic–pituitary–thyroid axis (HPT) regulates the production and inhibition of thyroid hormones under normal conditions. Situations such as severe illness cause dysregulation of the thyroid system termed non-thyroidal illness syndrome (NTIS), which causes the HPT axis to become allostatic [17]. Dysregulation of the fetal thyroid system causes devastating effects on maturation and survival because adequate levels of thyroid hormones are essential for proper fetal development [18]. Disruptions to the fetal pig thyroid system are especially detrimental to maturation because the placental enzymatic barrier restricts the transfer of potentially compensatory maternal thyroid hormone. Deiodinases contribute to the regulation of thyroid hormone metabolism in the pig fetus [16], and their expression is altered in a compensatory fashion during NTIS in humans [17]. Metabolism of maternal thyroid hormone into inactive rT3 and T2 in the placenta prevents the fetus from being overstimulated by active thyroid hormone. Deiodination of fetal T4 produces bioactive T3 to supply peripheral fetal tissues with adequate amounts of active thyroid hormone [19].

Deiodinases are not the only enzymes to have metabolic effects on thyroid hormone. Sulfotransferases modify thyroid hormone by catalyzing the addition of a sulfo group, which prevents the hormone from being deiodinated or acting on receptors [13]. In adults, sulfotransferases and sulfo tags are mainly used to mark thyroid hormones for excretion (Figure 1). However, in the fetus, sulfotransferases ensure that the thyroid hormone is in a dormant state until it is needed and acts as a reservoir of potentially bioactive hormone [20]. Sulfatase catalyzes the removal of the sulfo group placed by sulfotransferases, restoring the thyroid hormone to its original state, and stimulating the resumption of bioactive and metabolic action [21]. This pathway is a relatively unexplored component of metabolism but is known to vary in the fetus throughout gestation [13, 22].

We have previously observed a profound decrease in thyroid hormone following PRRSV infection at all stages of the production cycle [7, 23, 24]. To explore the cause of the abnormal fetal thyroid state, we evaluated thyroid metabolism within the maternal endometrium (END), fetal placenta (PLC), fetal kidney (KID), and fetal liver (LVR) in previously established fetal phenotypes. Examination of the fetal placenta and maternal endometrium allowed us to observe changes on both sides of the placental enzymatic barrier. We investigated the effects on the fetal liver and kidney to observe changes in thyroid hormone metabolism within the two major metabolic organs of the fetus [25]. To understand how viral infection altered the availability of bioactive thyroid hormone within these tissues, we concentrated on the expression of three iodothyronine deiodinases, five sulfotransferases, sulfatase, and two solute carriers known to transport thyroid hormone. Finally, we used liquid chromatography–tandem mass spectrometry (LC–MS/MS) to measure levels of thyroid hormone metabolites in fetal serum.

## Materials and methods

### Animal model

Fetal samples were obtained from a late gestation challenge model, which has been previously described [23]. In short, Yorkshire gilts (*n* = 27) bred to Landrace boars were housed at BSL2 animal facilities at the University of Saskatchewan beginning ~ 78 days of gestation. At gestation day 86, gilts (*n* = 22) were inoculated with either 1 × 10^5^ TCID_50_ NVSL 97-7895 split evenly between intramuscular (2 mL IM) and intranasal (1 mL in each nostril) inoculation. The remaining gilts (*n* = 5) were sham inoculated in a similar manner with minimum essential media (MEM) and serve as gestational age matched controls (CON). Gilts and fetuses were euthanized at 21 days post-infection (dpi) with a total of 30 mL of sodium pentobarbital diluted 1:1 with sterile water. Roughly, 50% was initially delivered IV into the vena cava, and once the gilts were deeply sedated and in lateral recumbence, the remaining dose was delivered via intra cardiac injection. All fetuses exhibiting an umbilical pulse were categorized as live (*n* = 286) and further classified based on the presence (MEC) or absence (VIA) of meconium staining. For each VIA and MEC fetus, a segment of the maternal–fetal interface adjacent to the umbilical stump was isolated, and the fetal portion of the placenta (PLC) was peeled away from the maternal epithelium. The uterine myometrium was then removed to yield samples of the endometrium (END). In addition, fetal blood was collected from the axillary artery, and samples of the central portion of the fetal kidney (KID) and the outer most tip of the left lobe of fetal liver (LVR) were snap frozen using liquid nitrogen. In total, tissue samples were collected from 83 CON fetuses and 203 PRRSV-challenged fetuses immediately following euthanasia. All animal work was conducted in strict accordance with the guidelines of the Canadian Council of Animal Care and with the approval of the University of Saskatchewan’s Animal Research Ethics Board (Protocol #20180071).

### Sample selection

Viral load was assessed in fetal serum and thymus as previously described [26]. In short viral RNA was isolated QIAamp Viral RNA and RNeasy (Qiagen Inc. Toronto, Ontario, CA, USA) column based methods as per the manufacturers instruction. Viral copy number was then determined by qPCR relative to a standard curve. This data was then used in conjunction with fetal preservation to identify previously established fetal phenotypes. These classifications consisted of control from sham inoculated gilts (CON), uninfected with no detectable viral load (UNIF), high viral load viable (HV-VIA), or high viral load with severe meconium staining (HV-MEC) with both high viral groups showing > 5 log_10_ of virus in serum and thymus. Circulating T4 was then evaluated in all fetuses as previously described [7] and *n* = 10 fetuses per group with the lowest within-group Z score for T4 were selected for further investigation.

### Absolute quantification of gene expression

Analysis of gene expression was conducted in adherence to the MIQE guidelines as outlined in Bustin et al. [27]. Samples of END, PLC, KID, and LVR were finely ground under liquid nitrogen using a mortar and pestle, and total RNA was extracted using a double precipitation protocol [28] with TRIzol (Thermofisher, Waltham, MA, USA). The total RNA was quantified using a Nanodrop ND-1000 (ThermoFisher), and the integrity was established using electrophoresis on denaturing agarose gels [29], with all samples showing clear 28 S and 18 S bands in a roughly 2:1 ratio. An aliquot of 20 µL of each total RNA sample was DNase treated using the Turbo DNA-free Kit (Invitrogen) and 0.5 µL of RNaseOUT (ThermoFisher). Then 2 µg per sample of DNase-treated RNA was reverse transcribed using the High-Capacity cDNA Reverse Transcription Kit (Applied Biosystems, Foster City, CA, USA). The samples were diluted to yield 10 ng/µL cDNA, and the libraries were stored at − 20 °C. Specific primers targeting genes of interest were designed using current RefSeq mRNA sequences (Table 1) to cover all predicted transcript variants. Primers were identified using the BLAST-like alignment tool (BLAT) relative to the Sus Scrofa 11.1 assembly and designed to span exon-exon junctions. Initial primer efficiency was determined to be within the range of 95–105% for each target and melting curve analysis displayed no evidence of multiple amplicon products. Validated primers were amplified using qPCR on a CFX Connect Real-Time System (Bio-Rad, Hercules, CA, USA) in tissues with established expression to produce the target amplicon product. The PCR product was then prepared using the TOPO TA Cloning Kit (Invitrogen, Carlsbad, CA, USA) as described by the manufacturer and transfected into chemically competent *Escherichia coli* (TOP10). Positive transfectants were identified on kanamycin selection plates, cultured in equivalent Luria broth (LB), and plasmid purified using GeneJet Plasmid Miniprep Kit (Thermofisher) as described by the manufacturer. A 6-point standard curve starting at 10^8^ copies/µL with 1:10 serial dilution was made for each target, and a stringent efficiency range of 95–105% was verified using qPCR as previously described [30]. Absolute quantification PCR was completed using 10 ng cDNA in duplicate, including standard curve, with SsoAdvanced Universal SYBR Green SuperMix (Bio-Rad) on a CFX Connect Real-Time System (Bio-Rad). Gene expression is presented in the form of target transcript copy number per 20 µg equivalent cDNA [31, 32].

**Table 1:** **Gene specific primer sequences used for qPCR**

Pathway	Official symbol	Gene ID	Forward primer	Reverse primer	Annealing temp (°C)	Amplicon length (bp)	Target RefSeq
Deiondination	DIO1	414380	5′-ACTTCATGCAAGGCAACAGG-3′	5′-GGTCCTGGAGATTCTGGTGA-3′	61	213	NM_001001627.1
	DIO2	414379	5′-CTCGGTCATTCTCCTCAAGC-3′	5′-TCACCTGTTTGTAGGCATCG-3′	61	140	NM_001001626.2
	DIO3	414378	5′-CCTATCTGCGTGTCTGACGA-3′	5′-GCCTGCTTGAAGAAATCCAG-3′	61	92	NM_001001625.2
Sulfation	SULT1A3	396640	5′-GGTGTCCCACAGGTTTTGAG-3′	5′-CGACGTAGACCACCTTGACC-3′	61	123	NM_213765
	SULT1B1	100624541	5′-TGGCTGGAAATGTGGCTTAT-3′	5′-TCACCAGAGGGTTGTCCTTC-3′	61	228	XM_005656511
	SULT1C2	100623541	5′-GAAACCTCAGTCAGCGGAAA-3′	5′-TTCGGGTTCCTCTTGATGTC-3′	61	125	XM_013995894.2
	SULT1E1	397052	5′-GGGAGGAATTTTGGGTGACTA-3′	5′-GCCAGATTTGGGATAGGTGA-3′	61	204	NM_213992.1
	SULT2A1	641359	5′-CCAAGGAAATGTGCCCTATG-3′	5′-GTCCCGTTTCAGCTCTTCAT-3′	61	109	XM_005664644
	STS	448816	5′-AGACCCTCAGGACTCCCAAT-3′	5′-AAACTCCCACCTGGTTCTGA-3′	61	162	XM_021080518
Transportation	SLC16A2	100513513	5′-AGTGGAGTTCCAAGCAGCAT-3′	5′-AGCCCAAACGATCAGTGAAT-3′	61	95	XM_021080455.1
	SLC16A10	100513770	5′-CACCCATTGCAGGGTTACTC-3′	5′-TATGGAGCCAAGGGATGAAA-3′	61	117	XM_021091212.1

### LC–MS/MS analysis

To conserve valuable fetal sera, a subset (*n* = 6/group) of fetuses from those analyzed via qPCR (*n* = 10/group) were selected for analysis based on the lowest within-group Z-score for T4. In addition, a six-point standard curve containing 0.8–0.0001 ng/mL each of T4, rT3, 3,3′T2, and 3,5T2 (Cayman Chemical, Ann Arbor, MI, USA) was created and 50 µL of each standard was combined with 450 µL of serum substitute (2% BSA). Serum T4 and metabolites were assessed via LC–MS/MS using a previously established method [33]. T4 in serum was measured and compared to previous measurements to confirm validity of the new protocol. In short, an aliquot of 500 µL of fetal serum, or standard curve in serum substitute, was combined with 120 µL protection solution consisting of 25 mg/mL of ascorbic acid, citric acid, and dl-Dithiothreitol, along with 25 µL of L-^13^C_6_-T4 (1 ng/ µL) (Sigma-Aldrich, St. Louis, MO, USA) as an internal standard. Samples were then extracted with 1 mL of 100% acetone, mixed, and incubated on ice for 10 min before centrifugation at 2500×*g* for 2 min at 4 °C. The resulting supernatant was aspirated, and the remaining pellet was reextracted twice with 1 mL of 50% acetone. The combined supernatants were then reduced to 1 mL by evaporation under a steady stream of compressed nitrogen. The remaining liquid was combined with 1 mL of ddH_2_O and transferred to a solid-phase extraction column (Waters Corporation, Milford, MA, USA). The columns were centrifuged at 130×*g*, washed with 3 mL of 30% MeOH, and eluted with 3 mL of MeOH with 0.1% acetic acid. The elution was again evaporated to 1.5 mL under a nitrogen current and then placed in a Savant SpeedVac SC110 Concentrator, with no heat, to completely evaporate overnight. Before analysis, all samples were resuspended in 100 µL of MeOH + 0.1% acetic acid.

An Agilent 1290 Infinity II liquid chromatography (LC) system coupled to an Agilent 6470 series triple quad LC–MS/MS (Agilent Technologies, Santa Clara, CA, USA) was used to analyze all samples. A Waters HSS T3 2.1 mm × 100 mm, 1.8 μm column with a HSS T3 VanGuard 2.1 mm × 5 mm precolumn was used for LC separation (Water Corp, Milford, MA, USA). The buffers were water with 10 mM ammonium acetate and 0.1% acetic acid (solvent A) and methanol with 0.1% acetic acid (solvent B). The linear LC gradient was as follows: time 0 min, 40% B; time 1 min, 40% B; time 11 min, 80% B; time 16 min, 100% B; time 17 min, 40% B; time 25 min, 40% B. all at a constant flow rate of 0.25 mL/min. Data were acquired in positive electrospray ionization (ESI) mode. The jet stream ESI interface had a gas temperature of 325 °C, gas flow rate of 5 L/min, nebulizer pressure of 45 psi, sheath gas temperature of 250 °C, sheath gas flow rate of 7 L/min, capillary voltage of 3500 V in positive mode, and nozzle voltage of 500 V. The ΔEMV voltage was 300 V. Agilent Masshunter Quantitative analysis software was used for data analysis (version 10.1). Finally, the resulting hormones concentrations were converted to SI units (mmol/L).

### Statistical analyses

All data analysis was carried out in R 4.0.3 [34]. All data was analyzed non-parametrically with the Kruskal–Wallis test followed by the Wilcoxon rank-sum test for post-hoc comparisons. Gene expression was normalized as copy number per 20 ng of equivalent cDNA (CN/20 ng Eq cDNA) [35]. The resulting *P*-value was adjusted for multiple comparisons with the Bonferroni correction. Significant changes in gene expression levels within a given tissue are expressed as an absolute value of the percentage change between the median expression levels of the specified groups. The ggplot2 package [36] was used to visualize all data with significant statistical differences (*P* < 0.05) represented by unique superscripts.

## Results

### qPCR evaluation of fetal thyroid metabolic gene expression during PRRSV infection

**Figure 2:**
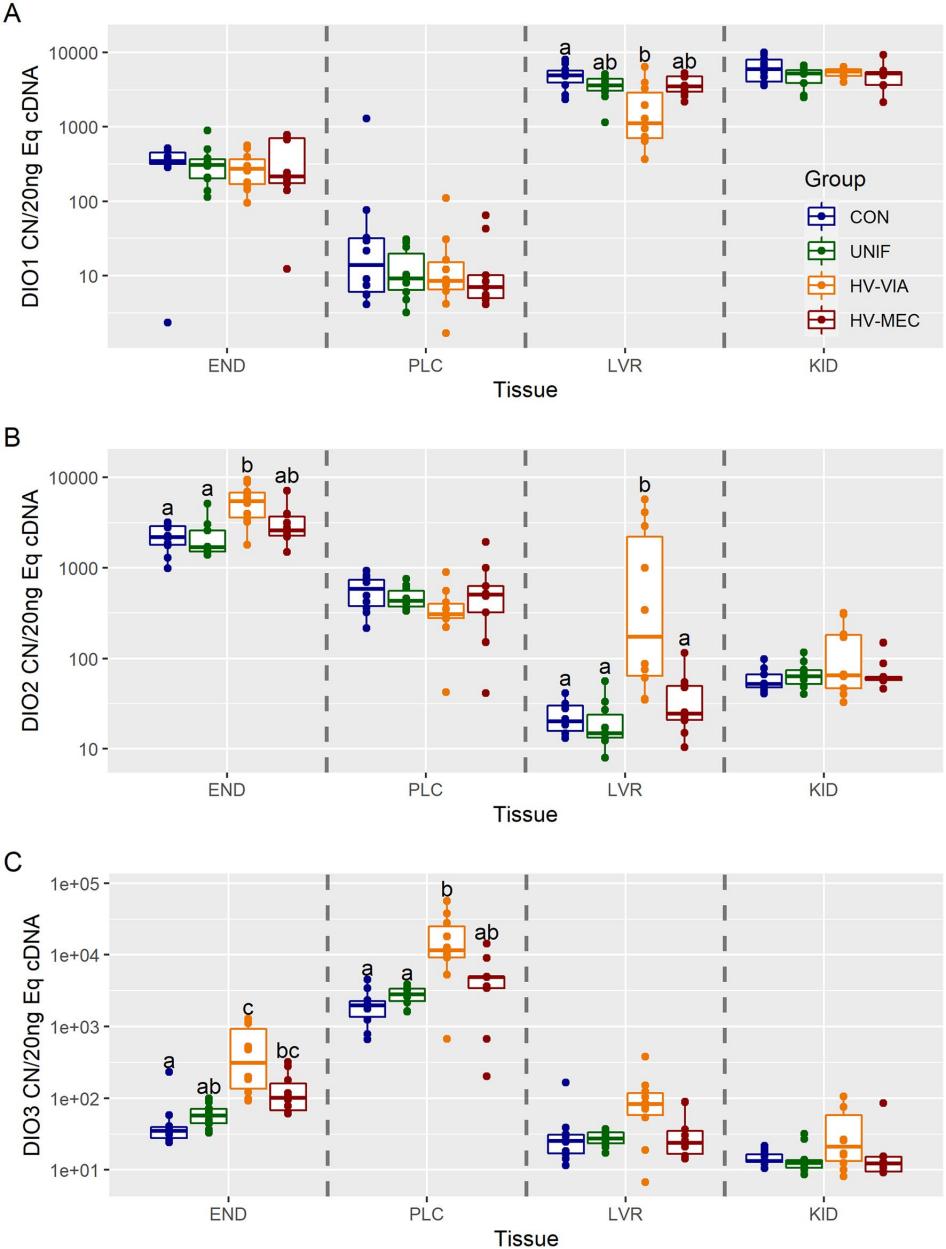
**Deiodinase gene expression.** Transcriptional abundance for DIO1 (**A**), DIO2 (**B**), and DIO3 (**C**) in maternal endometrium (END), fetal placenta (PLC), fetal liver (LVR), and fetal kidney (KID) derived from fetuses of Sham inoculated control (CON, *n* = 10) and PRRSV-2 challenged dams at 21 days post maternal infection. Fetuses from challenged dams were classified based on serum and thymus viral load as being uninfected (UNIF, *n* = 10), high viral load viable (HV-VIA, *n* = 10), or high viral load with meconium staining (HV-MEC, *n* = 10). Gene expression was evaluated using a standard curve generated from plasmid and normalized as copy number per 20 ng of equivalent cDNA to quantify gene expression in each group in each tissue with different superscripts denoting statistical differences between groups (*P* < 0.05) within a given tissue.

To assess potential changes in thyroid hormone metabolites, we first evaluated gene expression of deiodinases, which activate and deactivate thyroid hormones. There was no significant difference in expression of DIO1, 2, and 3 between CON and UNIF fetuses in the four evaluated tissues (Figure 2). Expression of DIO1 was the highest in the KID, followed by LVR and END, with minimal expression in PLC (Additional file 1). Expression was significantly downregulated by 77% in the fetal liver of the HV-VIA group relative to CON (*P* = 0.035) (Figure 2A). No significant differences in DIO1 expression were observed for the HV-MEC group in LVR. There was no difference in the expression of DIO1 across the four groups in END (*P* = 0.730), PLC (*P* = 0.732), or KID (*P* = 0.483). DIO2 had the highest median expression in the END followed by PLC, KID, and LVR. DIO2 expression was significantly upregulated by 964% in the LVR (*P* = 0.002) and 150% in the END (*P* = 0.008) of the HV-VIA group relative to CON (Figure 2B). This upregulation contrasts with DIO1, which had downregulated expression in the same group and tissue. Like DIO1, no significant difference in expression of DIO2 was detected in KID (*P* = 0.791) or PLC (*P* = 0.161). We evaluated DIO3 and found that the highest median expression was in the PLC, followed by END, LVR, and KID. DIO3 expression in the HV-VIA fetuses was significantly upregulated by 871% in END (*P* = 0.005) and 489% in PLC (*P* = 0.013) relative to CON (Figure 2C). Expression at the END of the HV-MEC fetuses was significantly upregulated by 190% from CON (*P* = 0.010) but did not significantly differ from UNIF (*P* = 0.187). No significant differences were observed in the PLC of the HV-MEC group. There was no significant difference in DIO3 gene expression in LVR (*P* = 0.075), and, like DIO1 and DIO2, no significant change was detected in KID (*P* = 0.212).

### Sulfation pathway expression

**Figure 3:**
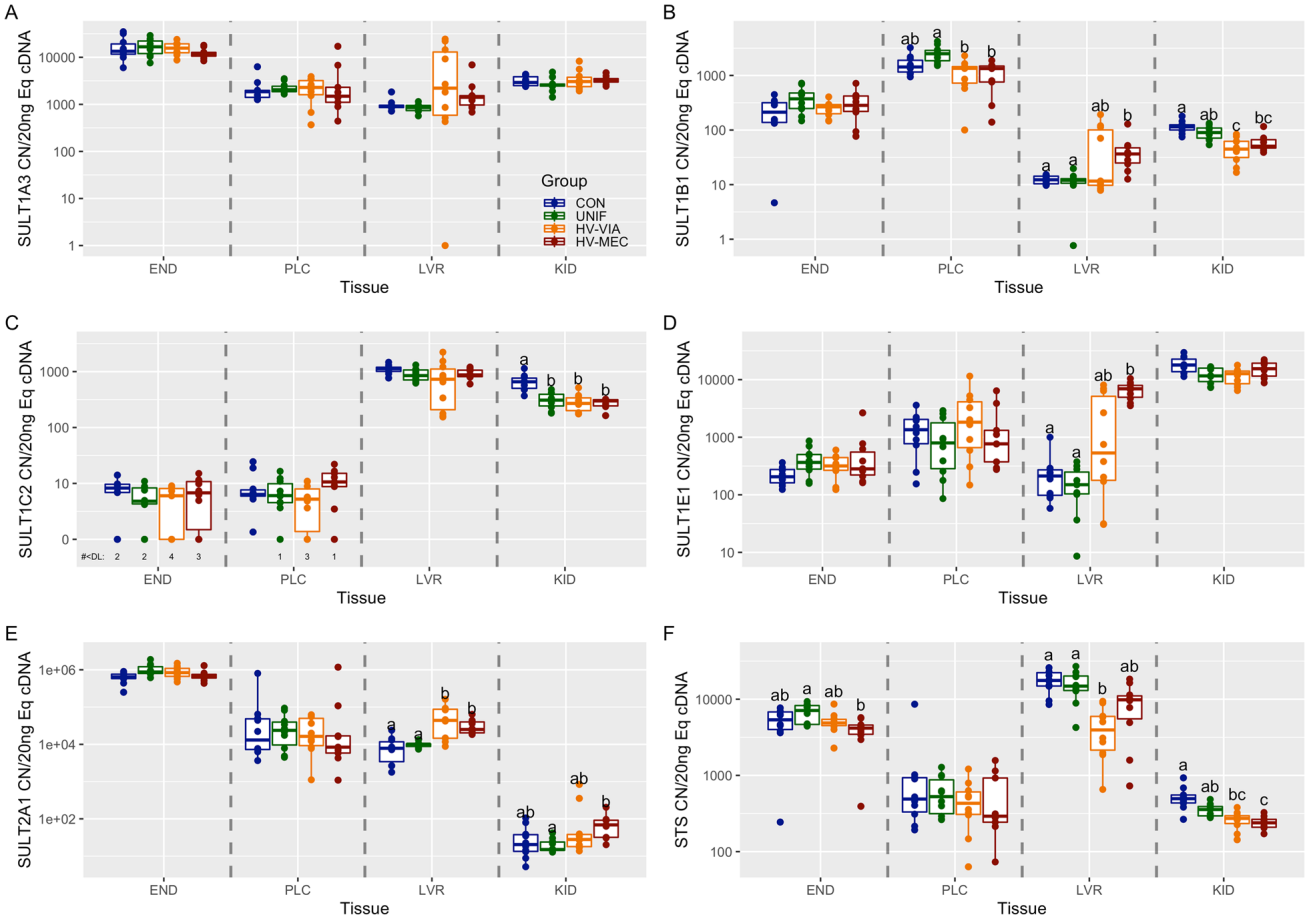
**Sulfotransferase gene expression.** Transcriptional gene expression for SULT1A3 (**A**), SULT1B1 (**B**), SULT1C2 (**C**), SULT1E1 (**D**), SULT2A1 (**E**), and STS (**F**) in maternal endometrium (END), fetal placenta (PLC), fetal liver (LVR), and fetal kidney (KID) derived from fetuses of Sham inoculated control (CON, *n* = 10) and PRRSV-2 challenged dams at 21 days post maternal infection. Fetuses from challenged dams were classified based on serum and thymus viral load as being uninfected (UNIF, *n* = 10), high viral load viable (HV-VIA, *n* = 10), or high viral load with meconium staining (HV-MEC, *n* = 10). Gene expression was evaluated using a standard curve generated from plasmid and normalized as copy number per 20 ng of equivalent cDNA to quantify gene expression in each group in each tissue with different superscripts denoting statistical differences between groups (*P* < 0.05) within a given tissue.

To evaluate changes in sequestration and targeted excretion, we next evaluated the expression of genes known to sulfate or disulfate thyroid hormones. There was no significant difference in the expression of SULT1A3, SULT1B1, SULT1E1, and SULT2A1 between CON and UNIF fetuses in the four evaluated tissues (Figure 3). The highest median expression of SULT1A3 was in END, followed by KID, PLC, and LVR (Additional file 1). Expression of SULT1A3 had no significant differences across the four phenotypes in any of the four tissues (END: *P* = 0.258, PLC: *P* = 0.540, LVR: *P* = 0.063, KID: *P* = 0.383) (Figure 3A). SULT1B1 had the highest median expression in PLC, followed by END, KID, and LVR. Expression of SULT1B1 was significantly decreased by 46% in the PLC of both HV-VIA (*P* = 0.006) and HV-MEC (*P* = 0.006) groups relative to UNIF but no significant difference relative to CON was observed for either group (Figure 3B). SULT1B1 expression was also significantly suppressed in the KID of the HV-VIA group by 60% (*P* = 0.002) and the KID of the HV-MEC group by 56% (*P* = 0.008) relative to CON. Interestingly, SULT1B1 expression was significantly upregulated by 197% in LVR of the HV-MEC group relative to CON (*P* = 0.005). There were no significant differences in SULT1B1 expression in END (*P* = 0.243). SULT1C2 had the highest median expression in LVR, followed by KID, with END and PLC showing near equivalent levels and approaching the limit of detection. SULT1C2 expression was significantly downregulated in the KID of UNIF by 52% (*P* = 0.003), HV-VIA by 58% (*P* = 0.003), and HV-MEC fetuses by 54% (*P* = 0.002) all relative to CON (Figure 3C). This is the only instance of the CON and UNIF groups being significantly different in this experiment. There were no significant differences in SULT1C2 expression in END (*P* = 0.420), PLC (*P* = 0.177), or LVR (*P* = 0.131). SULT1E1 had the highest median expression in KID, followed by PLC, with LVR and END showing near equivalent levels. Expression of SULT1E1 was significantly upregulated in LVR of the HV-MEC group by 3145% relative to CON (*P* = 0.001) and 4531% relative to UNIF (*P* = 0.001) (Figure 3D). SULT1E1 expression in the LVR of the HV-VIA group was not significantly different from the other groups. There were no significant differences of SULT1E1 expression between groups in END (*P* = 0.108), PLC (*P* = 0.562), or KID (*P* = 0.022). The final gene evaluated in the SULT family, SULT2A1, had the highest median expression level in END followed by PLC, LVR, and KID. SULT2A1 had significantly upregulated expression by 459% in LVR of the HV-VIA (*P* = 0.01) and 219% in HV-MEC (*P* = 0.003) relative to CON (Figure 3E). Interestingly, upregulation of SULT2A1 was noted in the KID of HV-MEC but only relative to UNIF (*P* = 0.01). There were no significant differences of SULT2A1 expression between groups in END (*P* = 0.020) or PLC (*P* = 0.779). Finally, we evaluated the expression of STS and found the highest median expression was in LVR, followed by END and PLC with near equivalent levels, and then KID. STS expression was affected in three out of the four evaluated tissues (Figure 3F). Like many other evaluated genes in this experiment, there were no significant differences in STS expression between the CON and UNIF groups. Expression of STS was significantly decreased in the END of HV-MEC by 41% relative to UNIF (*P* = 0.022) but not CON, which was similarly seen in KID SULT2A1 expression. STS was significantly downregulated by 76% in LVR (*P* = 0.002) and 45% in KID (*P* = 0.006) of HV-VIA fetuses relative to CON. Still, significant suppression in HV-MEC fetuses relative to CON was only noted in KID (*P* = 0.003). There were no significant differences in STS expression in PLC (*P* = 0.731).

### Transporter gene expression

**Figure 4:**
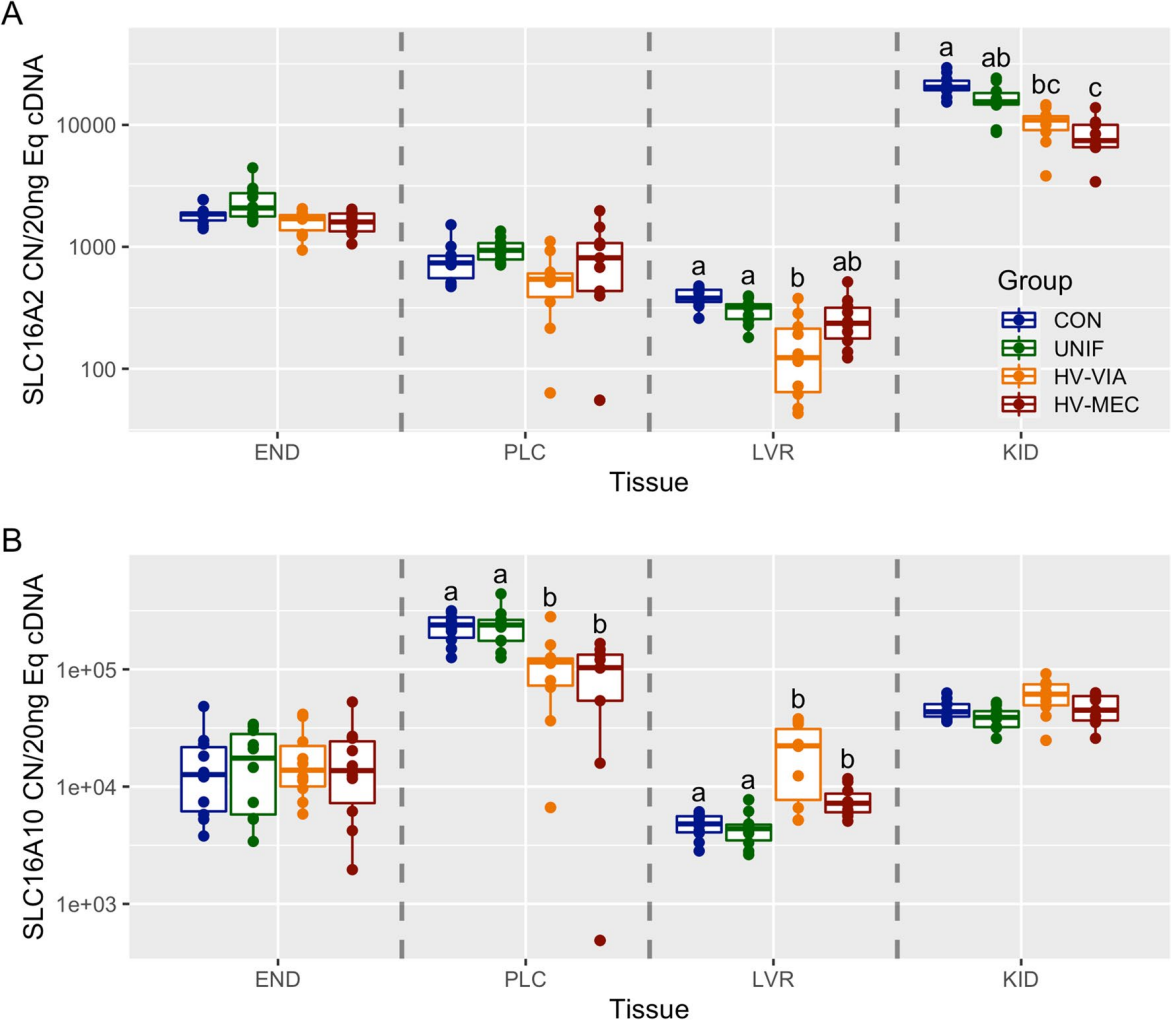
**Gene expression of canonical thyroid transporters.** Transcriptional abundance of SLC16A2 (**A**) and SLC16A10 (**B**) in maternal endometrium (END), fetal placenta (PLC), fetal liver (LVR), and fetal kidney (KID) derived from fetuses of Sham inoculated control (CON, *n* = 10) and PRRSV-2 challenged dams at 21 days post maternal infection. Fetuses from challenged dams were classified based on serum and thymus viral load as being uninfected (UNIF, *n* = 10), high viral load viable (HV-VIA, *n* = 10), or high viral load with meconium staining (HV-MEC, *n* = 10). Gene expression was evaluated using a standard curve generated from plasmid and normalized as copy number per 20 ng of equivalent cDNA to quantify gene expression in each group in each tissue with different superscripts denoting statistical differences between groups (*P* < 0.05) within a given tissue.

To evaluate changes in transit of thyroid hormones, we next evaluated the expression of two canonical transporters, SLC16A2 and SLC16A10. There was no significant difference in expression of SLC16A2 or SLC16A10 between CON and UNIF fetuses in the four tissues evaluated. The highest median expression of SLC16A2 was in KID followed by END, PLC, and LVR (Additional file 1). Expression of SLC16A2 was significantly decreased in KID of HV-VIA fetuses by 56% (*P* = 0.001) and by 63% in HV-MEC fetuses (*P* = 0.002) relative to CON (Figure 4A). SLC16A2 expression was also significantly downregulated by 67% in the LVR of HV-VIA fetuses (*P* = 0.005) relative to CON, with the HV-MEC group approaching significance (*P* = 0.055). There were no significant differences in SLC16A2 expression between groups in END (*P* = 0.030) or PLC (*P* = 0.063). Finally, when evaluating SLC16A10, we found that the highest median expression was in PLC followed by KID, END, and LVR. Expression of SLC16A10 was significantly downregulated in PLC of the HV-VIA group by 51% (*P* = 0.017) and 56% in the HV-MEC group (*P* = 0.006) relative to CON (Figure 4B). However, expression was upregulated by 362% in LVR of the HV-VIA group (*P* = 0.010) and 50% in the HV-MEC group (*P* = 0.010) relative to CON. There were no significant differences in SLC16A10 expression between groups in END (*P* = 0.953), or KID (*P* = 0.030).

### Serum thyroid hormones and metabolites

**Figure 5:**
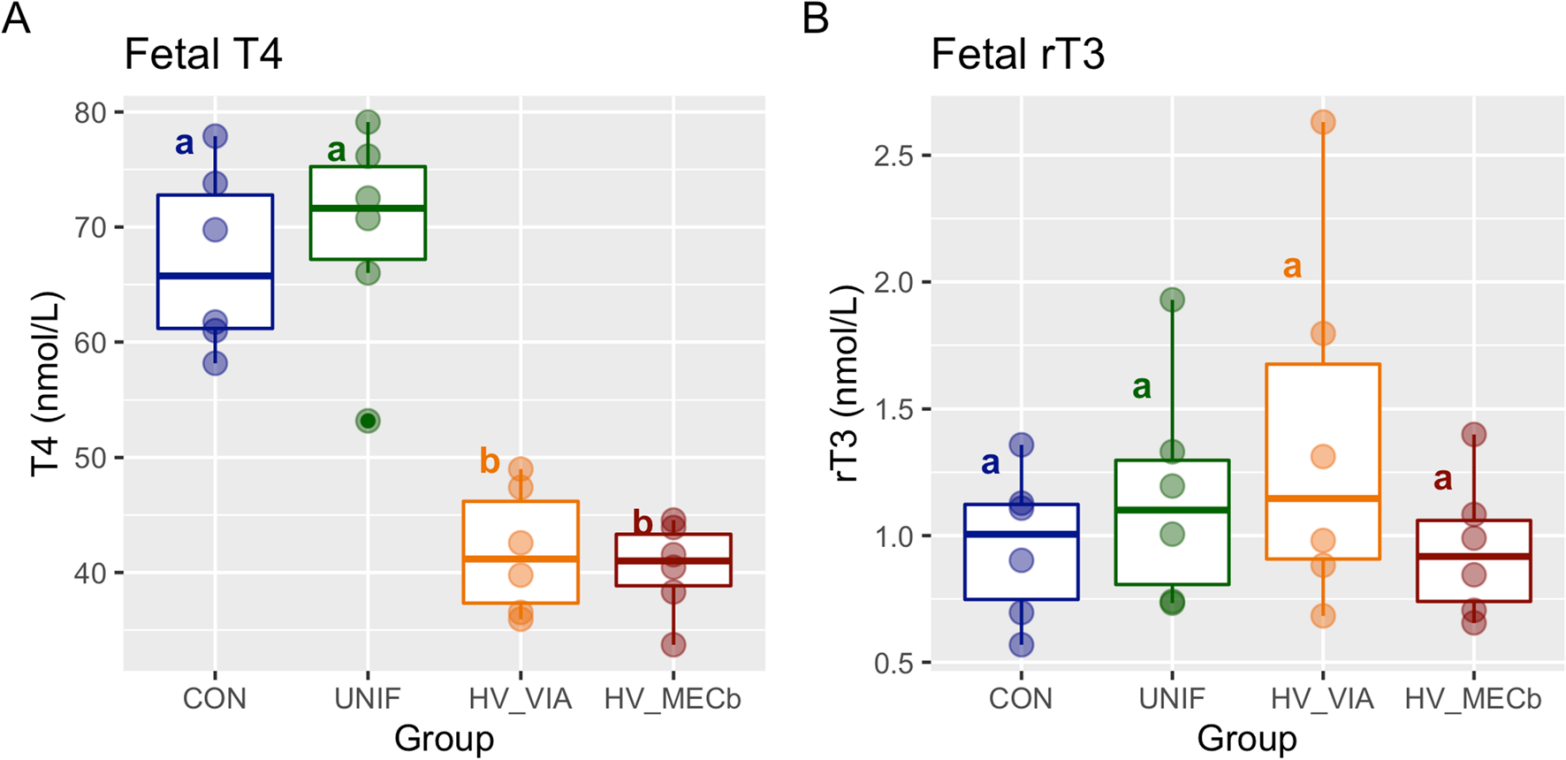
**Serum Thyroid hormone.** Boxplot of thyroxine (T4) (**A**) and reverse-triiodothyronine (rT3) (**B**) measured in fetal serum using liquid chromatography tandem mass spectrometry (LC–MS/MS) and converted to SI unit (nmol/L). Fetuses of Sham inoculated control (CON, *n* = 6) and PRRSV-2 challenged dams at 21 days post maternal infection. Fetuses from challenged dams were classified based on serum and thymus viral load as being uninfected (UNIF, *n* = 6), high viral load viable (HV-VIA, *n* = 6), or high viral load with meconium staining (HV-MEC, *n* = 6). Different superscripts denoting statistical differences between groups (*P* < 0.05).

To investigate systemic changes in fetal thyroid hormone metabolism, we quantified T4 and three inactive metabolites in fetal serum using liquid chromatography–tandem mass spectrometry. There was a significant difference in T4 levels in both high viral groups relative to the CON and UNIF (*P* < 0.001) (Figure 5A). However, there was no significant difference in rT3 across the four groups (*P* = 0.65) (Figure 5B). The two other metabolites measured (3,3′T2 and 3,5T2) were undetectable at a threshold of 0.0001 ng/µL.

## Discussion

Late gestation PRRSV infection has disastrous effects on the developing fetus during a critical window of terminal development. In addition to significant fetal losses, piglets born alive experience high levels of postnatal mortality [4, 37]. While the exact mechanism by which PRRSV disrupts terminal development is unknown, previous studies have demonstrated significant decreases in serum T3 and T4 in fetuses in highly infected fetuses [7, 23] during a period when both hormones are known to rapidly increase in the healthy fetus [15]. Interestingly, T3 levels differed between fetuses classified as viable and those exhibiting meconium staining, with the latter maintaining higher levels of circulating T3 despite the equivalent viral load. The observed fetal hypothyroidism was then suggested to be independent of maternal thyroid function. Therefore, disruption in fetal thyroid hormones must result from either dysregulation of the fetal HPT-axis or alteration in fetal thyroid hormone metabolism. This study investigated changes in gene expression involved in fetal thyroid hormone metabolism following PRRSV infection using absolute quantification. We then measured levels of thyroid hormone metabolites in fetal serum to determine if changes in expression of metabolic genes are associated with a concomitant shift in hormone levels.

The regulation of fetal development in mammals has been extensively studied in rodents and larger mammals such as sheep [13] and swine [38], whereas comparatively little is known about the fetal pig. Under normal conditions, both rat and human euthyroid fetuses express DIO1 and DIO2 at low levels in all tissues [22, 39]. However, we found that within the late gestation fetal pig, expression of DIO1 is most concentrated within fetal metabolic organs. At the same time, DIO2 is expressed at the highest levels at the maternal–fetal interface. Expression of these deiodinases increases in the late gestation of human fetuses, which is thought to increase fetal T3 production necessary for thermogenesis following parturition [40, 41]. Unlike DIO1 and DIO2, DIO3 is highly expressed in the human fetal placenta and liver [42–44]. This contrasts with our results in fetal swine, which show that DIO3 is most expressed at the maternal–fetal interface while at a low level in the fetal liver by comparison. However, this is consistent with DIO3 contributing substantially to the enzymatic barrier to maternal thyroid hormones in humans, rats, and pigs [42, 45]. A previous report also measured high T4 and T3 levels in swine maternal tissue and high rT3 and 3-3′-T2 in fetal tissue, concluding that high DIO2 and DIO3 activity must be present in the fetal portion of the pig placenta [16]. Our observations of DIO2 and DIO3 expression levels were consistent with these results, as both genes had the highest expression levels within maternal–fetal interface tissues. Collectively, we have developed an expression profile of deiodinases in four tissues critical to the development of the late gestation swine fetus. More critically, we found that the location of deiodinase expression in the normal pig fetus fundamentally differs from that previously established in humans and rats.

Although significant changes in the expression of genes involved in thyroid hormone metabolism were observed in all investigated tissues during fetal PRRSV infection, significant differences between groups were more common in the fetal liver and kidney than at the maternal–fetal interface. This suggests that fetal hypothyroidism following PRRSV infection is influenced more by metabolism within fetal organs than by the function of the placental enzymatic barrier. Expression of deiodinases in adult and fetal rat tissues has been shown to undergo a compensatory change in response to hypothyroidism, with increased DIO1 and DIO2 and decreasing DIO3 [46, 47]. We found that late gestation pig fetuses experiencing hypothyroidism but had no signs of compromise, showed decreased DIO1 expression in the liver, which could result in a local suppression of T4 conversion to active and inactive forms due to the dual activity of the enzyme. Interestingly, DIO3 was upregulated or trending towards upregulation in all tissues of the HV-VIA fetuses. This is inconsistent with the expected compensatory response and could result in increased levels of inactivated hormone metabolites. We also observed a concomitant upregulation of DIO2 expression in the liver of the HV-VIA group, which may increase the local availability of T3 at the expense of global T4 availability. While we identified the fetal liver as a leading site of alteration of thyroid hormone metabolic gene expression, these changes following PRRSV infection are not always consistent with changes in other species experiencing hypothyroidism.

An enzymatic barrier, created by the activity of deiodinases, is present early in gestation in the fetal pig placenta. This barrier, mediated by the presence of DIO2 and DIO3, metabolizes maternal thyroid hormones at the maternal–fetal interface to reduce transmission to the fetus. The limited need for maternal thyroid hormones is presumably associated with the development and function of an autonomous thyroid gland early in gestation [15]. There is evidence that the enzymatic barrier is modulated during changes in fetal thyroid status, such as during development and following thyroid disruptions [16]. As expected, expression of DIO3 within the human fetus and the placenta is suppressed during hypothyroidism to prevent active thyroid hormones from being deactivated and excreted [48]. Surprisingly, in the current study, the significant increase in DIO3 expression in the HV-VIA group suggests an increase in thyroid hormone degradation into deactivated forms within the fetal placenta and endometrium in response to infection. Such changes in the placenta would be consistent with the allostatic nature of NTIS, where the physiological set point for thyroid hormones is reduced, and normal homeostatic mechanisms are not activated.

The maternal–fetal interface is the point of exchange between the dam and fetus and is comprised of both fetal and maternal tissues. By separating these respective tissues, we were able to observe changes in the maternal compartment, which are dependent on fetal phenotype. Interestingly, expression of both DIO2 and DIO3 were found to be significantly upregulated in the endometrium, corresponding to the HV-VIA fetuses relative to the resistant UNIF fetuses. In this instance, the changes in uterine physiology are not driven by maternal infection or the associated immune response, as both HV-VIA and UNIF are derived from PRRSV-infected gilts. The specificity of this response to fetal phenotype suggests that the fetus can regulate maternal uterine physiology in a localized fashion. Similar regulation of the maternal endometrium by the porcine conceptus has been demonstrated early in pregnancy, where a combination of embryonic estrogens and interferons has been shown to upregulate STAT1 in the uterine tissue [49].

In the current study, we compared high viral load fetuses that were either susceptible (HV-MEC) or resilient (HV-VIA) to determine if changes in gene expression are associated with fetal viability. High levels of variation in gene expression seen in the HV-VIA group suggest that infection is a continuous process and indicates that some fetuses observed as viable at 21 dpi may, in fact, be on the brink of compromise. When comparing high viral groups, we observed that resilient fetuses had increased expression of liver DIO2 and maternal endometrium DIO3 relative to the susceptible HV-MEC fetuses. This suggests that the HV-MEC fetuses cannot upregulate deiodinase activity effectively during PRRSV infection, which is consistent with previous reports that individuals within this group respond to infection with physiologically distinct mechanisms [5, 8]. This finding may explain the previously noted decrease in circulating T3 in the susceptible fetuses [7, 23]. Viral infection has the potential to compromise exchange at the placenta through an increase in the expression of DIO3, which then decreases the amount of active maternal thyroid hormone that reaches the fetus. The fetus could have a compensatory response involving increasing DIO2 activity in attempt to increase internal T3 levels. The high deiodinase activity increases energy expenditure in the fetus, resulting in further stress and eventual death and suggesting that variation in fetal outcome may be associated with the ability of the fetus to upregulate DIO2 and DIO3 expression.

Sulfotransferases are pleiotropic, exhibiting a capacity to sulfate various target molecules, either altering bioactivity or tagging a substrate for excretion. From the existing literature, we have identified five human sulfotransferase genes with established activity on thyroid hormones (SULT1A3, SULT1B1, SULT1C2, SULT1E1, and SULT2A1) [22, 50–52], which have corresponding homologous genes in the pig. Sulfotransferases are more prominent in the human fetus relative to adults and act as a sink for fetal thyroid hormones, allowing for hormone sequestration and reutilization [13, 40]. Beyond that, not much is known about sulfotransferases in the human fetus, and even less is known about the function of these enzymes in pigs [22]. We found that SULT1E1 had the highest level in the fetal kidney, suggesting that this enzyme contributes to the generation of sulfated thyroid hormones within the pig fetus. Like SULT1E1, SULT1C2 was prominent in the fetal liver and kidney, which is consistent with previous studies [51]. There is evidence of sulfotransferase modification of maternal thyroid hormones of the rat at the placenta [52]. While SULT1C2 showed almost undetectable expression levels within placental tissues, SULT1A3, SULT1B1, and SULT2A1 are expressed at the highest levels at the maternal–fetal interface out of the four tissues studied. Although it is uncertain if these sulfotransferases actively sulfate maternal thyroid hormones or another compound, these enzymes, along with DIO2 and DIO3, may play a role in the enzymatic barrier at the pig placenta.

We also investigated sulfatase (STS), the enzyme responsible for removing sulfo groups placed by sulfotransferase, thereby reactivating dormant thyroid hormones. We observed that STS was most expressed in the maternal endometrium and within the fetal liver of the pig. Previous observations of hypothyroid fetal sheep and rats show amounts of dormant sulfated T3 decrease over time, presumably converted back into active T3 by similarly increasing amounts of STS [53–55]. However, we found that STS expression is significantly decreased in PRRSV-infected fetal tissues. This suggests that sulfated reserves of fetal thyroid hormones may not be utilized by the fetus to compensate for the observed systemic hypothyroidism.

To determine if the observed changes in gene expression manifest in systemic changes, we used liquid chromatography–tandem mass spectrometry to evaluate serum T4 and its non-bioactive metabolites. In reevaluating T4 by this method, we have confirmed the profound decrease in both high viral groups reported previously [7]. We expected inactive metabolite levels in the serum to be increased because of increased DIO3 gene expression in multiple tissues. However, rT3 levels are not different in the high viral groups compared to CON and UNIF. This may suggest that the effect of changes in deiodinase expression is localized to the respective tissue. Alternatively, the lack of an increase in circulating rT3 may result from the reduced availability of its T4 precursor. This is further supported by the lack of detection of 3,3′T2, which would similarly be derived from T3, which was also suppressed in these fetuses [7].

Collectively, our results show that PRRSV infection causes dysregulation in genes involved in fetal thyroid hormone metabolism within the fetus and at the placental enzymatic barrier to maternal thyroid hormones. Notably, the fetus appears to locally regulate maternal gene expression in the endometrium after fetal infection. Fetal viability is associated with the upregulation of DIO2 and DIO3 expression, suggested by the differences between the HV-VIA and HV-MEC fetuses. Finally, instances of sulfotransferase, sulfatase, and transporter protein gene expression dysregulation are evident in infected fetuses, regardless of preservation status. In terms of thyroid hormone metabolism, the fetuses fail to compensate for lack of available thyroid hormones during PRRSV infection, but rather show signs of decompensation. From this study, it is unclear if fetal decompensation is derived from viral infection or if it is a biological protective mechanism. Our findings suggest that a non-infective model of fetal hypothyroidism could be useful in distinguishing the pathophysiology of viral infection from fetal thyroid disruption. This model would be valuable in investigating the direct impact of hypothyroidism on fetal development and the potential compensatory response in the placenta and within the fetus.

## Supplementary Information

**Additional file 1. Raw absolute quantification gene expression data. **Median copy number per 20 ng equivalent cDNA in maternal endometrium (END), fetal placenta (PLC), fetal liver (LVR), and fetal kidney (KID) tissues that were derived from fetuses of Sham inoculated control and PRRSV-2 challenged dams at 21 days post maternal infection. Color indicates median expression values of a given gene within a given tissue, with the highest value being coded green and lowest coded red within gene.

## Abbreviations

CON: control fetuses collected from mock-infected gilts
dpi: days post-infection
END: maternal endometrium
HV-MEC: meconium-stained fetuses from PRRSV infected gilts with greater than 5 log_10_ at 21 dpi and 4.5 log_10_ at 12 dpi of virus in both fetal serum and thymus
HV-VIA: viable fetuses from PRRSV infected gilts with greater than 5 log_10_ at 21 dpi and 4.5 log_10_ at 12 dpi of virus in both fetal serum and thymus
KID: fetal kidney
LVR: fetal liver
NTIS: non-thyroidal illness syndrome
PLC: fetal portion of the placenta
PRRSV: porcine reproductive and respiratory syndrome virus 2 (Strains NVSL 97-7895 and KS-2006-72109)
rT3: reverse triiodothyronine
T2: diiodothyronine
T3: triiodothyronine
T4: thyroxine
UNIF: viable fetuses from PRRSV infected gilts with no detectable virus in either fetal serum or thymus
