# Supplementary figures and images for: Effects of porcine reproductive and respiratory syndrome virus (PRRSV) on thyroid hormone metabolism in the late gestation fetus
Source: Vet Res. 2022 Sep 30;53:74. doi: 10.1186/s13567-022-01092-3 (PMC9524047; doi:10.1186/s13567-022-01092-3)

## Slide 1
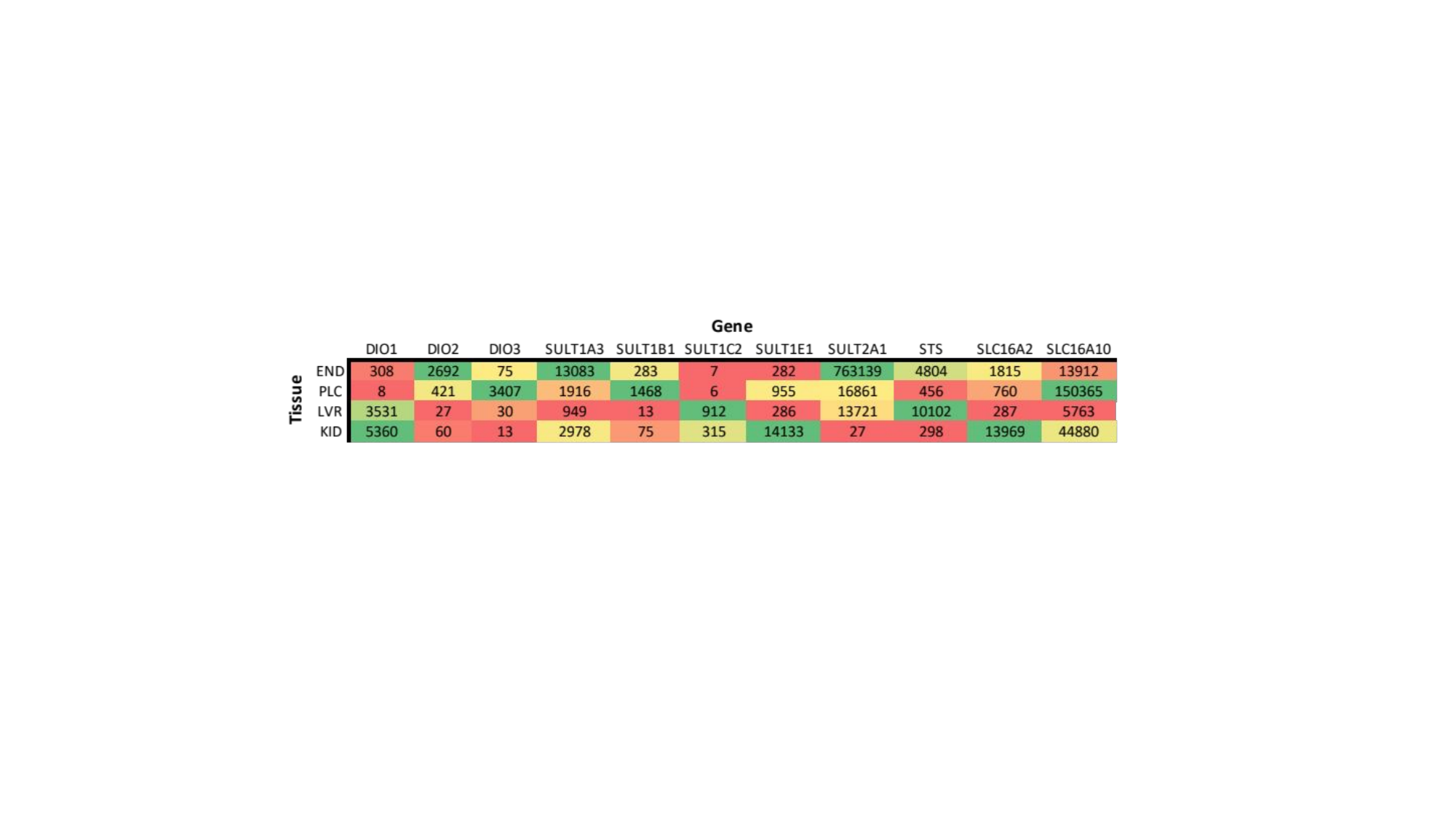

Supplement: Supplementary file 1 — Additional file 1. Raw absolute quantification gene expression data. Median copy number per 20 ng equivalent cDNA in maternal endometrium (END), fetal placenta (PLC), fetal liver (LVR), and fetal kidney (KID) tissues that were derived from fetuses of Sham inoculated control and PRRSV-2 challenged dams at 21 days post maternal infection. Color indicates median expression values of a given gene within a given tissue, with the highest value being coded green and lowest coded red within gene. [file 13567_2022_1092_MOESM1_ESM.pptx]
